# Living happily ever after: longitudinal insights into enduring mental health and wellbeing

**DOI:** 10.1007/s00127-026-03134-z

**Published:** 2026-06-03

**Authors:** Lianne P. de Vries, Christel M. Middeldorp, Meike Bartels

**Affiliations:** 1 Department of Biological Psychology, Vrije Universiteit Amsterdam, Van der Boechorststraat 7, Amsterdam, 1081 BT The Netherlands; 2 Amsterdam Public Health Research Institute, Amsterdam University Medical Center, Amsterdam, The Netherlands; 3 Department of Child and Adolescent Psychiatry and Psychology, Amsterdam UMC, Amsterdam, The Netherlands; 4 Arkin Mental Health Care, Amsterdam, The Netherlands; 5 Academic Center for Child and Adolescent Psychiatry, Levvel, Amsterdam, The Netherlands

**Keywords:** Mental health, Wellbeing, Longitudinal, Genetic, Risk and resilience factors

## Abstract

**Purpose:**

*Enduring Mental Health* (EMH) reflects maintaining good psychological functioning without developing mental health problems. Estimates of the proportion of individuals who have EMH vary widely. We investigated EMH across the lifespan, defined not only as absence of mental health problems, but also as consistent presence of high wellbeing. To explore how defining EMH affects the phenotype, we included both stricter and broader lenient thresholds for mental health problems and low wellbeing.

**Methods:**

Data came from 16,030 Netherlands Twin Register participants (35% male; mean birth year 1970) with ≥ 4 assessments over 8–20 years. For each EMH definition, preregistered analyses included computation of prevalence, genetic and environmental influences using twin modeling, and phenotypic and genetic correlates including education, personality, social factors and polygenic scores.

**Results:**

Threshold choice for low mental health strongly affected prevalence of EMH: 71% (absence of problems),93% (high wellbeing), and 71% (combined) for stricter thresholds versus 42%, 82%, and 42% for lenient thresholds of low mental health. Heritability (46–61%) and patterns of associations were similar across definitions. Optimism, social support, and good self-rated health were positively associated with EMH (OR = 1.5–2.9), while neuroticism, loneliness, and stress lowered the likelihood (OR = 0.3–0.8).

**Conclusion:**

Defining EMH using different thresholds for mental health changed prevalence estimates, whereas heritability and correlates were broadly similar across definitions. Threshold choice is important for estimating how common EMH is, while the consistent pattern of correlates suggest shared underlying mechanisms that can inform prevention to help individuals maintain mental health.

**Supplementary Information:**

The online version contains supplementary material available at https://doi.org/10.1007/s00127-026-03134-z.

## Background

Mental health problems are common, but estimates vary substantially depending on whether they refer to diagnosable disorders, clinically relevant symptoms, or broader experiences of psychological distress. Findings from the World Mental Health Surveys across 29 countries suggest that approximately half of the population meet criteria for a mental disorder at some point in their lives [39]. Broader estimates that also include milder or subclinical symptoms suggest that lifetime experiences of mental health problems may be considerably more common, with some estimates as high as 80% to 90% [52]. These findings suggest that maintaining good mental health across long periods of life may be relatively uncommon. This phenomenon has been referred to as *Enduring Mental Health* (EMH; [53]), typically defined as the long-term absence of mental health problems across repeated assessments.

Mental health includes more than just the absence of symptoms such as depression or anxiety; it also includes the presence of positive components of wellbeing. Dual-factor models propose that positive and negative aspects of mental health are related but distinct constructs, such that individuals may experience relatively high wellbeing despite symptoms, or low wellbeing in the absence of substantial mental health problems [25, 34, 65]. Wellbeing can broadly be defined as feeling good and functioning well, and is considered a multidimensional construct that includes both cognitive and affective components, such as life satisfaction and positive affect [21, 22]. These indicators of wellbeing are strongly related, both phenotypically and genetically [4, 7, 30, 35]. In the current study, we used life satisfaction, reflecting the cognitive-evaluative component of wellbeing.

Previous research on EMH has mainly focused on the enduring absence of mental health problems [3, 19, 52, 53, 55], thereby neglecting the positive side of mental health. In the present study, we build on this work by broadening the operationalization of EMH, defined not only by the consistent absence of mental health problems, but also by the consistent presence of a positive indicator of mental health, namely wellbeing. Note that we do not consider the absence of EMH using this strict classification to be equivalent to poor mental health. Experiencing mental health symptoms or lower wellbeing temporarily to some degree is a normal part of life, particularly in response to adversity or major life events, and may coexist with good functioning or resilience. This stricter definition of EMH is intended to identify a subgroup characterized by continued optimal mental health over time.

Although the mechanisms underlying EMH remain insufficiently understood, previous work suggests that it may reflect a combination of lower vulnerability to psychopathology and the presence of protective personal and environmental resources. The broader operationalization of EMH may help identify factors associated not only with the absence of disorder, but also with sustained wellbeing, thereby complementing broader dual-factor models in which wellbeing and symptoms may co-occur.

Empirical evidence on EMH is relatively scarce. In a longitudinal study following children and adolescents from 9 months to 14 years old, 41% did not develop mental health problems, i.e., defined as no parent- or self-reported difficulties above the abnormal cutoff of the Strengths and Difficulties Questionnaire (SDQ), and were classified as having EMH [19]. These children demonstrated higher cognitive ability, strong emotion regulation, cooperation, enjoyment of school, and had mothers with no history of mental health problems [19]. Similarly, Alrouh et al., [3] found that 37% of children in the Netherlands Twin Register followed from ages 3 to 12 showed EMH, using a subclinical symptom cutoff of the Child Behavior Checklist (CBCL). In addition to higher academic achievements and parental education, children with EMH reported higher wellbeing. 54% of the variance in EMH was due to genetic factors, and genetic influences on EMH were observed through polygenic scores related to education and mental health.

The emergence of most psychiatric disorders during adolescence and young adulthood [56] indicates the importance of studying EMH beyond childhood across the lifespan. As expected due to a longer measurement period, these studies reported a lower prevalence of EMH, compared to rates found in children. In a longitudinal sample (*n* = 988) tracking individuals from ages 11 to 38, only 17% of participants showed EMH, determined based on diagnostic interviews [53]. These individuals shared several protective factors, including better childhood self-control, a larger social network, no family history of mental illness, higher education, and higher-quality relationships. Similarly, using diagnostic interviews, in a 20-year study of adults from midlife to late adulthood (*n* = 964, baseline mean age = 35.4), 26% were classified as having EMH [55]. Correlates of EMH included higher educational attainment, larger social networks, lower neuroticism, no family history of mental health problems, and being employed.

The variation in EMH prevalence across studies (17%-41%) likely reflects differences in both sample composition and the way mental health problems are defined. Importantly, studies have differed in whether they operationalized mental health problems as diagnosable disorders, clinical-level difficulties, or subclinical increases in symptoms. These definitions are not directly comparable. When mental health problems are defined narrowly as diagnosable disorders, more individuals are likely to be classified as free of problems. In contrast, when mental health problems are defined more broadly to include mild or subclinical symptoms, fewer individuals will meet criteria for EMH. This distinction is important when interpreting apparently low EMH prevalence estimates: such estimates do not necessarily imply that most individuals develop a psychiatric disorder, but may instead reflect that many people experience some degree of symptoms at one or more points in life. Differences in thresholds may therefore influence prevalence estimates, which individuals are classified as having EMH, and influence whether similar predictors and correlates are observed across studies. Comparing stricter and more lenient thresholds for low mental health therefore allows us to examine whether EMH findings are robust across alternative operationalizations, or whether different thresholds capture meaningfully different groups.

Here, we investigate EMH across the lifespan using a definition of mental health that includes both the consistent absence of mental health problems and the consistent presence of high wellbeing. We also compare alternative thresholds for mental health problems and wellbeing to examine whether prevalence estimates, predictors, and correlates of EMH are robust across definitions. Based on previous literature, we expected prevalence estimates of EMH to vary depending on the thresholds used, with lower prevalence for broader definitions of mental health problems that include subclinical symptoms. We further expected EMH to be associated with more favorable psychosocial characteristics, including lower neuroticism, loneliness, and stress, and higher optimism, social support, and self-rated health. Finally, we explored whether the pattern of genetic and psychosocial correlates would be similar across definitions.

## Methods

### Participants and procedure

Participants are voluntary members of the Netherlands Twin Register, established over 30 years ago by the Department of Biological Psychology, Vrije Universiteit Amsterdam [37]. The NTR collects longitudinal survey data about lifestyle, personality, psychopathology, and wellbeing in twins and their families. A total of 57,206 participants completed at least one survey during adolescence and/or adulthood, including mental health and/or wellbeing measures. Participants have completed up to 11 surveys over a span of 8 to 20 years, with assessments every two to four years. The present study included 16,030 participants with data on these variables from four or more surveys (see supplementary Table S1). This selected subsample included 35% males and the average birth year was 1970, with a range of 1922–1999). Comparisons between the full and selected subsample showed small but significant differences (more females, older age, higher wellbeing, fewer mental health problems in the subsample; *d* = 0.01–0.19; *p* < .001), indicating minimal selection bias (see Table 1). To evaluate potential bias due to selective survey completion, we conducted sensitivity analyses. Individuals with slightly higher wellbeing were more likely to complete more surveys (β = 0.02, *p* = 1.09 × 10⁻¹⁰), and similarly, those with slightly higher anxious-depressed symptoms also tended to complete more surveys (β = 0.11, *p* = 1.06 × 10⁻¹⁵, see supplementary Figure S1 and Table S2).

**Table 1:** Differences between the total sample of participants in the NTR and the subsample of participants that completed four or more surveys that included data on mental health and/or wellbeing

	≥ 1 survey	≥ 4 surveys	Difference test (t)	*p*-value	Effect size (d)
*N*	57,206	16,030			
Sex (% males)	39.7%	35.2%	X^2^=103.0	**< 0.001**	h = 0.09
Year or birth	1973	1970	21.8	**< 0.001**	0.19
Range	1909–2004	1922–1999			
Wellbeing	7.70 (1.0)	7.78 (0.86)	-8.48	**< 0.001**	0.07
Range	0–10	1–10			
*Mental health problems*					
Anxious-Depressive	4.79 (4.65)	4.85 (4.36)	-1.58	0.114	0.01
Range	0–36	0–31			
Attention	4.64 (3.43)	4.47 (3.22)	5.44	**< 0.001**	0.05
Range	0–26	0–25			
Aggression	3.09 (2.91)	2.77 (2.48)	13.28	**< 0.001**	0.12
Range	0–34	0–20			
Intrusive	1.69 (1.74)	1.62 (1.58)	4.56	**< 0.001**	0.04
Range	0–12	0–11			
Rule Breaking	2.15 (2.01)	1.87 (1.60)	16.96	**< 0.001**	0.14
Range	0–30	0-16.5			
Somatic	2.74 (2.59)	2.65 (2.37)	3.93	**< 0.001**	0.04
Range	0–23	0–18			
Thoughts	1.64 (1.97)	1.36 (1.51)	17.78	**< 0.001**	0.15
Range	0–23	0–14			
Withdrawn	2.35 (2.30)	2.27 (2.02)	3.9	**< 0.001**	0.04
Range	0–17	0–15			

### Measures

For an overview of all measures and details, see supplementary Table S3.

#### Mental health problems

Mental health problems were assessed with the ASEBA Youth Self-Report (YSR) or Adult Self-Report (ASR; [1]), covering eight syndrome scales. These syndrome scales include Anxious/Depressed (18 items), Withdrawn (9 items), Somatic Complaints (12 items), Aggressive Behavior (15 items), Rule-breaking Behavior (14 items), Intrusive Behavior (6 items), Attention Problems (15 items) and Thought Problems (10 items). Participants rate the occurrence of the behavior, now or within the past 6 months, on a 3-point scale (0 - not true, 1 - sometimes true, and 2 -very true or often true). Example item are “*I am unhappy*,* sad*,* or depressed*”, “*I have trouble sitting still*”, and “*I get in many fights*”. The scores for the scales were computed by summing the items, with a higher score reflecting more symptoms. Internal consistency of the scales is good with α = 0.72-0.88, with a somewhat lower Cronbach’s alpha of 0.51 for Thought Problems [1].

Per syndrome scale and time point, sex- and age-specific *T*-scores were computed [1]. A T-score > 69 on any syndrome scale indicated mental health problems according to the *clinical* stricter threshold. A T-score > 65 on any syndrome scale indicated mental health problems at the *subclinical (or borderline)* lenient threshold.

####  Wellbeing

Wellbeing was assessed with the Dutch version of Cantril’s Self-Anchoring Striving Scale [16]. With a single item, participants have to indicate how they rate their lives in general, ranging from 1 (worst possible life) to 10 (best). To parallel the mental health thresholds, wellbeing was dichotomized as high vs. low using two cut-offs: strict (≥ 7 vs. ≤6 out of 10) and lenient (≥ 6 vs. ≤5 out of 10).

#### Enduring Mental Health (EMH)

We defined EMH as having (1) zero instances of mental health problems, (2) zero instances of low wellbeing, and (3) zero instances of mental health problems and low wellbeing, across all available surveys for a given participant, with a minimum requirement of four completed surveys. Participants who reported mental health problems and/or low wellbeing at any point during their participation were not classified as having EMH.

### Phenotypic correlates

The NTR is a longitudinal study in which participants completed surveys during adolescence and every few years in adulthood. When available, we included phenotypic measures from both adolescence and adulthood. For adulthood, if multiple assessments were available, we used the most recent score. Adolescent measures were considered predictors and adult measures outcomes of EMH. Additional information on all measures available in the Netherlands Twin Register can be found in the data showcase (https://ntrdata.nl/data-showcase.html).

#### Educational attainment

 Participants’ highest attained level of education was based on repeated self-reports collected across study waves. These were classified as: primary education only (1), lower vocational or secondary school (2), intermediate vocational, intermediate or higher secondary school (3) and higher vocational school and university (4).

####  Experience of stressful life events

 The number of experienced life events was assessed with an adapted version of a Dutch life-event scale (Schokverwerkings Inventarisatie Lijst [62]). Participants were asked whether they had experienced specific life events, and if so, when: *not at all*,* in the past year*,* in the past 5 years*, or *more than 5 years ago*. The sum of life events experienced were calculated for adolescence and adulthood separately. We grouped life events into 11 categories: including death of a partner, death of a child, death of another close one, serious illness of yourself, serious illness of a close one, divorce/end of a long term relationship, sexual crime, serious traffic accident, violent crime, job loss, and financial problems. Responses across surveys were reorganized to create a binary indicator per event, reflecting whether the participant had ever experienced that specific event.

#### Self-esteem

 Self-esteem was assessed with the Rosenberg Self-esteem scale [51]. The scale includes 10 items. Participants rate how strongly they agree or disagree with the items on a scale from 1 (Strongly Agree) to 4 (Strongly Disagree). An example item is “*I feel that I have a number of good qualities.*”

#### Neuroticism

 Neuroticism was measured using the NEO Five-Factor Inventory (NEO-FFI [17]), including 12 items which participants rated agreement with on a 5-point Likert rating scale from 1 (*strongly disagree*) to 5 (*strongly agree*). An example is “*I often feel tense and jittery*”.

####  Loneliness

Loneliness was assessed using 3 items of the short scale for loneliness [23, 28]. Participants rated how often they identify with the items on a scale from 1 to 3 (1 = hardly ever, 2 = some of the time, 3 = often). An example item is “*I feel isolated*”.

#### Self-rated health

Self-rated health was measured in both adolescence and adulthood with a single item ‘*In general*,* how would you rate your health*?‘. Participants rated their health on a five point scale ranging from 1 = “bad”, 2 = “mediocre”, 3 = “reasonable”, 4 = “good”, to 5 = “excellent”.

#### Exercise

Physical exercise was assessed broadly in both adolescence and adulthood with the question “*Do you exercise regularly*?”, resulting in a binary variable (yes/no).

#### Social support

The Duke–UNC Functional Social Support Questionnaire (FSSQ) was used in adults to assess the extent to which people give help and support when needed [14]. Participants rated the 8 items on a 5-point scale, from 1=‘much less than I would like’ to 5=‘as much as I would like’. An example item is “*I have people who care what happens in my life*”.

In adolescence, we used family functioning as a proxy, using the General Functioning subscale of the Family Assessment Device (FAD [41]). Participants rated 12 items on a 4-point scale, from 1=‘strong agreement to 4=‘strong disagreement’. An example item is ‘*In times of crisis we can turn to each other for support*.’

####  Optimism

 Optimism was assessed with the revised Life Orientation Test (LOT-R) [54]. Participants had to rate their agreement with 10 items on a 5-point Likert scale (1 = *strongly disagree*, 5 = *strongly agree*). We summed the scores on the three optimism items to create an optimism score. An example item is “*In uncertain times*,* I usually expect the best*”.

### Polygenic scores

Poygenic scores (PGS) are a measure of an individual’s genetic probability to develop a certain disorder or have a certain trait [66]. Using GWAS summary statistics, the PGS of a phenotype can be calculated in an independent sample by summing all genotype scores (at individual single-nucleotide polymorphisms) for a person after weighting them by their estimated effect size. The PGS of the phenotype can be used to test the predictive value towards another trait, or to investigate the shared genetic etiology between traits [47].

We used PGS to investigate whether and to what extent genetic risk for various mental health traits is associated with EMH. PGS were constructed using summary statistics from recent GWAS of phenotypes related to mental health and wellbeing. PGS were constructed using LDpred 0.9 with the infinitesimal model. PGS analyses controlled for 10 principal components and genotyping platform to account for population stratification.

The following PGS were included in our analyses: the wellbeing spectrum, including life satisfaction, positive affect, neuroticism, and depressive symptoms [6], Major Depressive Disorder [27], Educational Attainment [36], ADHD [20], Aggressive Behavior [29], Antisocial Behavior [58], Anxiety [48], Autism Spectrum Disorder [26], Bipolar Disorder [42], Childhood Maltreatment [64], Loneliness (Neale Lab, 2020), Neuroticism [43], PTSD [45], Risky Behavior [32], and Schizophrenia [59].

### Statistical analyses

Analyses were preregistered at https://osf.io/kp97s.

#### Prevalence of enduring mental health

We compared prevalence estimates across the three EMH definitions and two thresholds using cross-tabulations.

#### Etiology of enduring mental health

Genetic and environmental influences were estimated with classical twin models, using the OpenMx package [10] in R, on a subset of twins. Monozygotic (MZ) twin pairs share (nearly) 100% of their genes, whereas dizygotic (DZ) twin pairs share on average half of their genes. Using this difference in genetic overlap, the observed variance of traits can be decomposed into genetic and environmental variance components [12]. Genetic variance components include additive genetic variance (A) and non-additive or dominant genetic variance (D). Environmental variance consists of a common environmental variance component (C) (variance shared by family members) and a non-shared environmental component (E) that also includes measurement error. A choice for an ADE or ACE model is made based on the pattern of twin correlations. We applied a threshold model with one threshold for the binary EMH outcomes. Variance components (A, C/D, E) were tested using log-likelihood ratio tests.

#### Correlates of enduring mental health

Univariate logistic regressions with Generalized Estimating Equations (GEE; [40]) examined associations between EMH (1 = EMH, 0 = not) and 18 phenotypic variables or 15 PGSs. PGS analyses controlled for 10 principal components and genotyping platform to account for population stratification. Predictors significant in univariate models were entered into multivariate GEE models. Significance was set at α < 0.05 and corrected for multiple testing [9]. For the univariable regression analyses, p-values were corrected separately for the strict and lenient threshold analyses, with each correction based on 93 tests (31 predictors × 3 outcomes). For the multivariable regression analyses, correction was applied separately within each model set based on the number of predictors tested across the three outcomes.

### Deviation from preregistration

A deviation from the preregistration concerned the operationalization of Enduring Mental Health. In addition to the preregistered stricter clinical threshold for mental health problems, we included a more lenient subclinical/borderline threshold for mental health problems. Specifically, sex- and age-specific T-scores were computed for each syndrome scale at each time point, with T-scores > 69 indicating mental health problems according to the stricter clinical threshold and T-scores > 65 indicating mental health problems according to the more lenient threshold. Similarly, wellbeing was dichotomized as high vs. low using two cut-offs: strict (≥ 7 vs. ≤6 out of 10) and lenient (≥ 6 vs. ≤5 out of 10). These additional thresholds were included because closer review of the literature revealed heterogeneity in how Enduring Mental Health has been operationalized across studies. Including both thresholds for mental health allowed us to examine the robustness of the findings to the chosen cut-off and improved comparability with previous work.

## Results

### Prevalence of enduring mental health

Using a *stricter* threshold for mental health problems (i.e., only more severe problems count as problems), 71% of the sample never experienced any mental health problems and was classified as having EMH, whereas 93%, consistently reported high levels of wellbeing. When combining both criteria, i.e., absence of mental health problems and high wellbeing, 71% met the definition of EMH (see supplementary Table S4). Cross-tabulations showed that 22% of participants consistently reported high wellbeing but did report mental health problems at some point. The reverse pattern, no mental health problems but low wellbeing at any point, did not occur in the sample (see supplementary Table S5).

Using the *lenient* threshold for mental health problems (i.e., also borderline/subclinical problems count as problems), 42% of participants were classified as having EMH (i.e., never crossing the borderline threshold for mental health problems), whereas 82% consistently reported high wellbeing. Combining problems and wellbeing, 42% met the criteria for EMH (see supplementary Table S6). Cross-tabulations showed that 46% of participants reported consistently high wellbeing but did experience mental health problems at some point (see supplementary Table S7).

### Etiology of enduring mental health

Twin correlations (supplementary Table S8) indicated substantial genetic contributions to EMH. Across all models, the AE model (additive genetic and non-shared environmental influences) provided the best fit to the data (see supplementary Table S9 and S10). Using the *stricter* threshold, the heritability of EMH, defined as the absence of mental health problems, was estimated at 50% (95% CI: 38–60%). For wellbeing, the heritability was 61% (95% CI: 43–75%). When combining both the absence of mental health problems and the presence of wellbeing, the heritability was 51% (95% CI: 35–64%). Using the *lenient* threshold, heritability estimates were similar, i.e., 46% (95% CI: 35–56%) for mental health problems, 52% (95% CI: 38–63%) for wellbeing, and 46% (95% CI: 31–59%) for the combined phenotype.

Exploratory bivariate twin models between EMH based on problems and EMH wellbeing show genetic correlation of 0.91 and 0.80 and environmental correlation of 0.99 and 0.97 respectively for the strict and lenient threshold of problems or low wellbeing (see Supplementary Table S11 for all results). This indicates strong genetic and environmental overlap between the different operationalizations of EMH.

### Correlates of enduring mental health

#### Stricter threshold

**Fig. 1:**
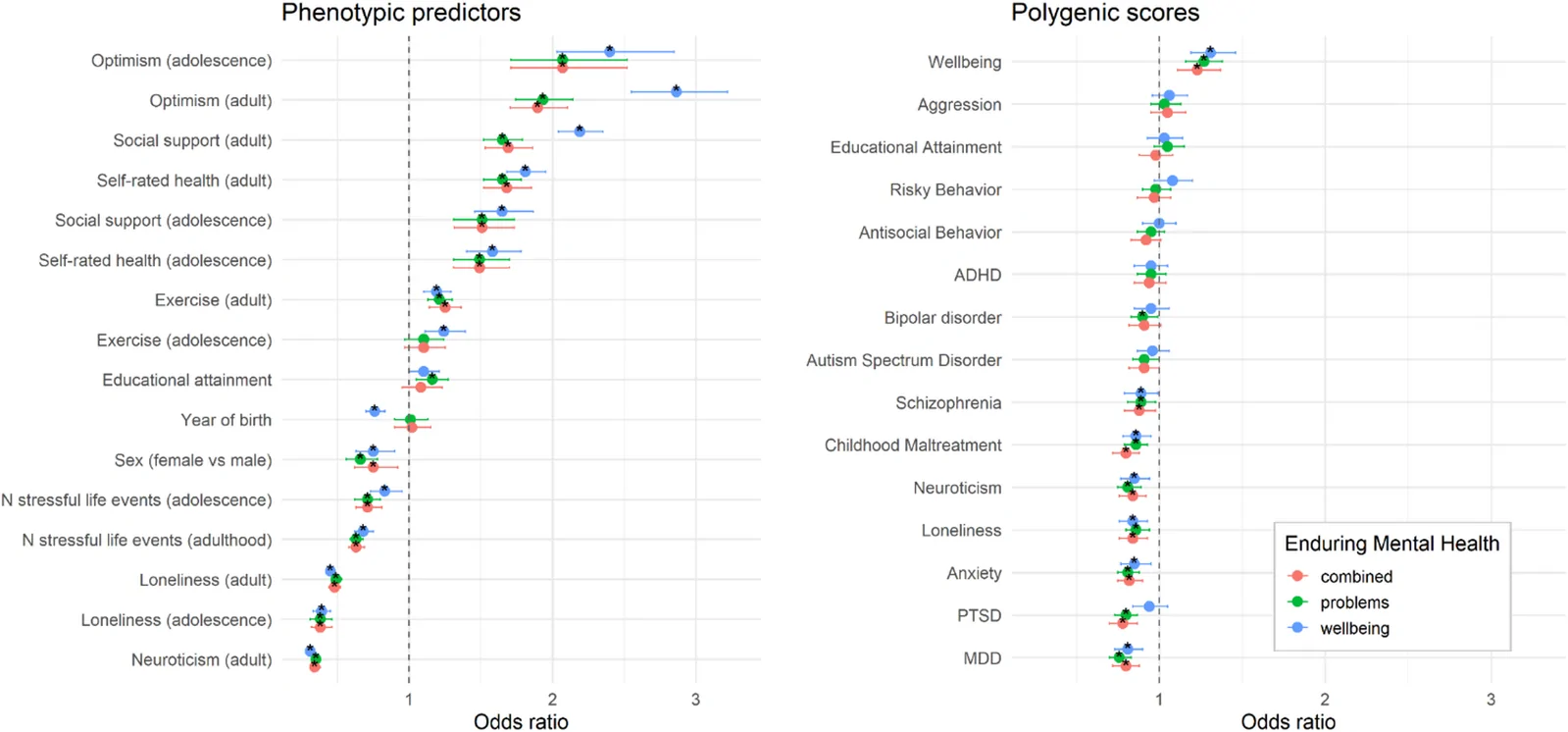
Correlates of Enduring Mental Health using a stricter threshold*. (adolescence) and (adult) indicate that the correlate is assessed respectively during adolescence and adulthood

Univariate regression models showed that enduring mental health was consistently associated with a range of factors across the three EMH phenotypes (absence of mental health problems, presence of wellbeing, and combined, see Fig. 1 and Supplementary Table S12). Patterns were largely similar across phenotypes and across adolescence and adulthood, indicating that the same traits can act as predictors and outcomes of EMH.

Psychological traits showed the strongest associations. Optimism in adolescence and adulthood were positively related to EMH (OR = 1.89–2.86), with the strongest effect for wellbeing (OR = 2.86, 95% CI = 2.55–3.22) compared to absence of problems (OR = 1.93, 95% CI = 1.74–2.14). Neuroticism showed strong negative associations across all phenotypes (OR = 0.31–0.68), indicating that emotional instability increases risk for poor mental health and lower wellbeing.

Social and contextual factors were also important. Adult social support related more strongly to enduring wellbeing (OR = 2.19, 95% CI = 2.04–2.35) than to absence of problems (OR = 1.65, 95% CI = 1.52–1.78). Loneliness (OR = 0.38–0.49) and stressful life events (OR = 0.63–0.81) were consistent risk factors. Year of birth was negatively associated with enduring wellbeing (OR = 0.76, 95% CI = 0.63–0.90) but not with absence of problems (OR = 1.01, 95% CI = 0.90–1.13), suggesting younger generations may report less enduring wellbeing while their risk of mental health problems remains stable.

Self-rated physical health showed consistent positive associations across all EMH phenotypes (OR = 1.49–1.81), indicating that physical and mental wellbeing are closely linked. Finally, polygenic scores (PGS) for wellbeing were positively associated with EMH, whereas higher PGS for major depressive disorder, PTSD, anxiety, loneliness, neuroticism, and childhood maltreatment were negatively associated (ORs < 1), suggesting that genetic predispositions to both positive and negative mental health traits contribute to enduring mental health.

#### Lenient threshold

**Fig. 2:**
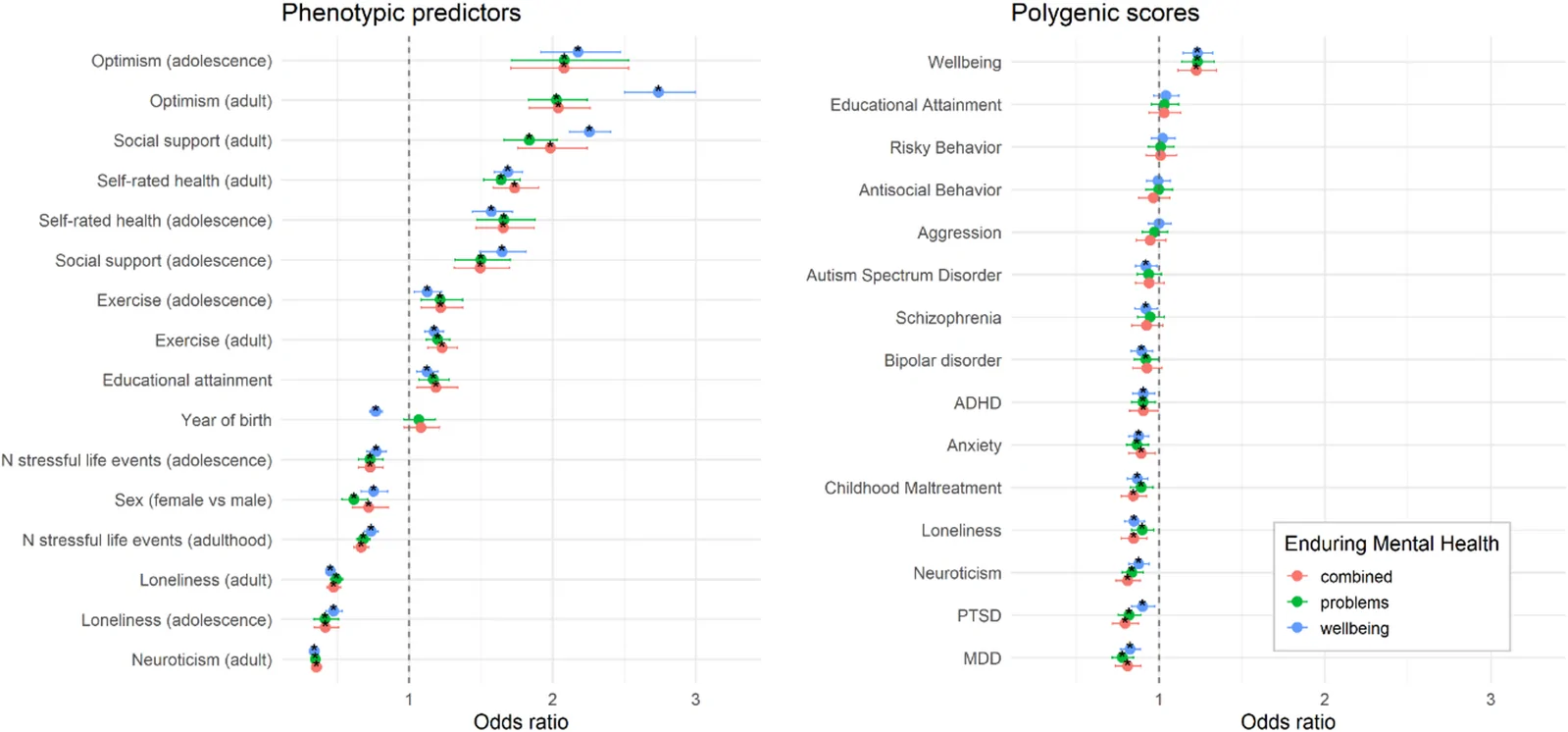
Correlates of Enduring Mental Health using a lenient threshold*. (adolescence) and (adult) indicate that the correlate is assessed respectively during adolescence and adulthood

Findings at the lenient threshold were largely consistent with those at the stricter threshold (see Fig. 2, supplementary Table S13, and for a direct comparison supplementary Figure S3 and S4).

### Multivariate regression models

Multivariate models of EMH were estimated in a substantially smaller subset of participants because these analyses required complete data on all predictors included in the model, including adolescent phenotypes, adult phenotypes, and polygenic scores. This resulted in an analysis sample of 277 participants for the model including both adolescent and adult predictors. To increase power, we additionally estimated a model including adult phenotypes only, which could be fitted in a larger sample of 1275 participants (Supplementary Table S14 and Figure S5).

Across models, neuroticism, loneliness and stressful life events (adolescent or adult) consistently predicted lower EMH. In the adulthood-only model, self-rated health also predicted EMH. Patterns were similar for mental health problems and wellbeing separately and for lenient thresholds, with social support and optimism specifically related to enduring wellbeing. Given small samples, estimates are imprecise.

## Discussion

Mental health includes both the absence of illness and presence of wellbeing. Research on Enduring Mental Health (EMH) should therefore capture both. Previous studies described EMH as relatively rare [3, 19, 53, 55]. We provide a more nuanced view by showing how methodological decisions affect prevalence. Using data from the Netherlands Twin Register, we found that EMH prevalence varied strongly with threshold choice, i.e., 71% for absence of mental health problems and 93% for high wellbeing (strict), versus 42% and 82% (lenient threshold). However, heritability estimates (~ 50%) and patterns of phenotypic and genetic associations were mostly consistent. Optimism, social support, and self-rated health were positively related to EMH and could be protective factors, whereas neuroticism, loneliness, and stress were negatively related to EMH and could be risk factors for mental health problems and low wellbeing.

### Definition of Enduring Mental Health

The concept of EMH was introduced by Schaefer et al., [53] to highlight that experiencing mental health problems at some point is more typical than exceptional. Reported prevalence across studies varies widely (17–41%), likely reflecting definitional differences [3, 19, 53, 55]. Our findings show that EMH prevalence strongly depends on mental health definitions and thresholds. Percentages across studies should therefore be carefully interpreted and definitional differences be taken into account.

Threshold choice has implications for how EMH is defined and interpreted. The stricter threshold for mental health problems resulted in higher EMH prevalence because it captured more severe symptoms, whereas the lenient threshold also classified milder or borderline symptoms as mental health problems. Which threshold is most appropriate depends on the research aim: stricter thresholds may be more suitable for identifying individuals free from clinically relevant problems, whereas more lenient thresholds may be preferable for capturing consistently optimal functioning and broader prevention-oriented outcomes.

We included a dual-factor perspective on enduring mental health, including both the absence of mental health problems and high wellbeing. More participants met criteria for enduring wellbeing than for absence of problems, and many reported high wellbeing despite symptoms (22% strict; 46% lenient), supporting the dual-factor model that wellbeing and mental health problems are related but not opposite ends of a single dimension [2, 5, 18, 25, 63, 65]. At the same time, we did not observe the reverse pattern of low wellbeing in the absence of symptoms. In our sample, and given our measure and operationalization of enduring wellbeing, this pattern may have been uncommon or not captured by our thresholds. Importantly, the wellbeing measure used in our study was life satisfaction, reflecting the cognitive-evaluative dimension of wellbeing, whereas wellbeing also includes other components, such as affective functioning. Although we might expect similar results with other wellbeing indicators due to their overlap, broader measures could result in different patterns. The distinction between negative and positive aspects of mental health may be particularly relevant in the context of stress, as stress may influence symptoms and wellbeing in partly different ways: some individuals may continue to evaluate their lives positively despite experiencing symptoms, whereas affective wellbeing might decline before more persistent mental health problems develop [63]. More broadly, our stricter EMH definition including both mental health and wellbeing is not intended to suggest that individuals classified with non-EMH are not functioning well, rather, we aimed to identify a subgroup with a particularly favorable and stable pattern of mental health over time and their predictors.

### Etiology and correlates of Enduring Mental Health

Despite variation in EMH prevalence, genetic architecture and psychosocial correlates were similar across definitions. Twin models showed substantial heritability (46–61%), with overlapping confidence intervals across definitions. Consistent with prior evidence, genetic and environmental factors influence both subclinical and clinical mental health to a similar extent [15, 33, 46, 57]. Our polygenic score analyses further indicate genetic liability relevant to EMH: polygenic scores for depression, anxiety, neuroticism, and related traits were associated with lower EMH, whereas the wellbeing polygenic score was associated with higher EMH.

Phenotypically, optimism, social support, and good self-rated health predicted EMH, while neuroticism, loneliness, and life stress predicted its absence. These findings align with resilience frameworks emphasizing internal and external resources that maintain wellbeing under stress [11, 24, 31, 38]. Social support, connection, and good physical health are frequently identified as resilience factors that buffer against stress and promote recovery [24, 31]. Furthermore, neuroticism, characterized by emotional reactivity and negative affectivity, may increase responses to challenges in life, increasing vulnerability to both poor mental health and reduced wellbeing. These findings also indicate that EMH is not only the absence of pathology, but reflects an active, adaptive process to stay mentally healthy, which is influenced by both stable personality traits, modifiable social-contextual factors, genetic effects, and potentially gene-environmental interplay.

A notable finding is the negative association between year of birth and enduring wellbeing (OR = 0.76), suggesting that younger cohorts are less likely to report high and stable wellbeing over time. This pattern was not observed for the absence of mental health problems (OR = 1.01), indicating that declines may be specific to positive aspects of mental health. While confidence intervals nearly overlap, the finding contributes to ongoing discussions on age, period, and cohort effects (e.g., [44, 50]), especially as research on generational trends in wellbeing remains limited. Some reviews report no increase in mental health problems over generations (e.g., [8, 49]), where others do, particularly in younger cohorts such as Millennials and Gen Z [13, 60, 61]. In our study, due to the need for four of more data points, younger generations were underrepresented (only ~ 2,000 of 16,000 born after 1990), but the associations between year of birth and EMH suggest that wellbeing may be more sensitive to societal changes than symptom-based mental health. One possible explanation is that wellbeing, particularly life satisfaction, may be more sensitive to societal change because it reflects people’s evaluations of their lives, potentially in relation to changing social, economic, and cultural conditions. This does not mean that problems are unaffected by such changes, but that differences may become visible earlier in wellbeing than in symptom-based indicators.Multivariate models confirmed that neuroticism, loneliness, and stressful life events remained significant predictors, with optimism and social support specific to enduring wellbeing. We broadly interpret adolescent correlates as potential predictors of EMH and adult correlates as possible outcomes. The similar patterns across adolescent and adult measures suggest bidirectional associations between risk and resilience factors and EMH. Further longitudinal and causal research is needed to disentangle shared etiology between EMH and other phenotypes and/or the directionality of these relationships.

### Limitations, implications, and future directions

A strength of this study is the use of a large longitudinal sample of over 16,000 participants, with data available from both adolescence and adulthood on a range of variables. This allowed us to examine EMH across developmental periods and assess both predictive and outcome-related correlates. However, this sample was a subset of the larger cohort of more than 52,000 individuals. While the subsample is mostly similar to the full cohort, there are some small differences in levels of wellbeing, depressive symptoms, and mental health, which may affect the results of this study. The sensitivity analyses to evaluate potential bias due to selective survey completion showed that individuals with slightly higher wellbeing were more likely to complete more surveys, but those with slightly higher anxious-depressed symptoms also tended to complete more surveys (see Methods and Figure S1). Although these effects were statistically significant due to the large sample size, the effect sizes were small, suggesting limited impact on the overall findings.

Furthermore, our methodological choice to use all available longitudinal data (ranging from 4 to 11 surveys) means that the observation period for being classified with a mental health problem varied across individuals. Participants with more completed surveys had more opportunities to report a mental health problem, which may have led to a lower absolute prevalence estimate of EMH compared to using a uniform, shorter observation period. However, we prioritized using all available data to prevent the misclassification of individuals who developed mental health problems later in the study, thereby capturing the ‘enduring’ nature of mental health as reliably as possible.

Additionally, only a limited subset of participants had complete data on all variables across adolescence and adulthood, which may reflect selective repeated participation and introduce bias in the results of the multivariate regression models. These analyses were based on complete cases and therefore have lower statistical power than the main analyses. We therefore need to interpret these multivariable findings with caution. Furthermore, the sample is a non-clinical population wide sample. Whereas there is variation in wellbeing and mental health problems, most participants in the Netherlands Twin Register are not in the clinical range. Findings should therefore be replicated in different samples, including diagnostic data. The largely similar findings across thresholds indicate that the correlates of EMH were not sensitive to variation in threshold choice. The direction and strength of associations were consistent and independent of the threshold. This indicates that threshold selection affects statistical classification but does not seem to alter the nature of enduring mental health, although replication is needed with diagnostic data of psychiatric diagnoses as well. If replicated, the consistency indicates the future use for both research and potential applied purposes. For example, the findings contribute to resilience research by identifying psychological (e.g., optimism), social (e.g., support), and biological (e.g., polygenic scores) factors that promote long-term mental health.

These results may also inform future research on prevention and mental health promotion. First, the findings indicate the value of positive psychological functioning alongside measures of mental health problems and provide insights in complete mental health across the lifespan. Second, optimism, social support, and physical health emerged as consistent correlates of EMH, suggesting that they may be important factors to consider in future studies aimed at understanding how enduring mental health is maintained over time.

## Conclusion

Enduring Mental Health can capture both the absence of mental health problems and the presence of wellbeing over time. In defining EMH, the threshold of mental health and wellbeing influences strongly the prevalence of EMH. However, there are consistent genetic and psychosocial factors that were associated to enduring mental health and wellbeing over time. Potential protective and risk factors include psychological traits, social factors, and genetic predispositions. These findings highlight the importance of a complete, dual-factor approach to understanding and promoting mental health across the lifespan.

## Electronic Supplementary Material

Below is the link to the electronic supplementary material.

Supplementary Material 1

## Data Availability

The data that support the findings of this study can be requested via the Netherlands Twin Register (https://ntrdata.nl/).

## Declarations

### Preregistration

The analyses of this study were preregistered (https://osf.io/kp97s).
