# Supplementary material for: Living happily ever after: longitudinal insights into enduring mental health and wellbeing
Source: Soc Psychiatry Psychiatr Epidemiol. 2026 Jun 3;61(11):2475–87. doi: 10.1007/s00127-026-03134-z (PMC13694541; doi:10.1007/s00127-026-03134-z)
Supplement: Supplementary file 1 [file 127_2026_3134_MOESM1_ESM.docx]

**Supplementary Material**

**Sample characteristics**

**Table S1.** Sample sizes and descriptives of EMH and predictor variables for the included sample.

|  | N | Mean | SD | Minimum | Maximum |
| --- | --- | --- | --- | --- | --- |
| Sex | 16025 | 1.65 | 0.48 | 1 | 2 |
| Year of birth | 16030 | 1969.74 | 16.38 | 1922 | 1999 |
| Education Attainment | 14392 | 3.41 | 0.76 | 1 | 4 |
| EMH problems | 3800 | 0.86 | 0.83 | 0 | 2 |
| EMH wellbeing | 8586 | 0.24 | 0.55 | 0 | 2 |
| EMH combined | 2662 | 0.86 | 0.83 | 0 | 2 |
| Self-rated health (adolescence) | 3456 | 4.23 | 0.66 | 1 | 5 |
| Self-rated health (adult) | 16027 | 4.04 | 0.72 | 1 | 5 |
| Self esteem (adolescence) | 3406 | 30.21 | 3.95 | 11 | 40 |
| Self esteem (adult) | 6217 | 31.65 | 4.24 | 10 | 40 |
| Optimism (adolescence) | 1730 | 21.08 | 3.22 | 6 | 30 |
| Optimism (adult) | 5582 | 16.06 | 3.57 | 0 | 24 |
| Neuroticism (adult) | 14446 | 29.68 | 7.88 | 12 | 59 |
| Loneliness (adolescence) | 1737 | 4.09 | 1.34 | 3 | 9 |
| Loneliness (adult) | 15548 | 3.83 | 1.2 | 3 | 9 |
| Exercise (adolescence) | 3470 | 0.81 | 0.4 | 0 | 1 |
| Exercise (adult) | 15899 | 0.65 | 0.48 | 0 | 1 |
| Social support (adolescence) | 3323 | 38.79 | 4.98 | 12 | 48 |
| Social support (adult) | 12782 | 36.34 | 5.02 | 8 | 40 |
| Number of stressful events (adolescence) | 3331 | 3.34 | 1.55 | 1 | 9 |
| Number of stressful events (adult) | 13865 | 3.90 | 1.79 | 1 | 10 |
| PGS Attention Deficit Hyperactivity Disorder | 10336 | -0.04 | 0.99 | -3.93 | 3.76 |
| PGS Aggression | 10336 | -0.04 | 0.98 | -3.78 | 3.78 |
| PGS Antisocial Behavior | 10336 | -0.03 | 0.98 | -3.38 | 4.35 |
| PGS Anxiety | 10336 | -0.01 | 1 | -3.74 | 3.70 |
| PGS Autism Spectrum Disorder | 10336 | -0.03 | 1 | -3.58 | 5.01 |
| PGS Bipolar | 10336 | -0.04 | 0.99 | -3.41 | 4.55 |
| PGS Childhood Maltreatment | 10336 | -0.03 | 1 | -3.59 | 4.46 |
| PGS Educational Attainment | 10336 | 0.09 | 0.98 | -3.29 | 3.85 |
| PGS Loneliness | 10336 | -0.03 | 0.99 | -3.94 | 3.81 |
| PGS Major Depressive Disorder (MDD) | 10336 | -0.05 | 0.98 | -3.60 | 3.79 |
| PGS Neuroticism | 10336 | -0.03 | 0.99 | -3.42 | 3.48 |
| PGS Post-traumatic stress disorder | 10336 | -0.04 | 0.95 | -3.72 | 8.81 |
| PGS Risky Behavior | 10336 | -0.02 | 1.01 | -3.61 | 3.62 |
| PGS Schizophrenia | 10336 | -0.08 | 0.97 | -4.02 | 4.85 |
| PGS Wellbeing | 10336 | 0.03 | 0.98 | -4.39 | 3.63 |

Note: EMH= Enduring Mental Health, PGS=polygenic scores.

**Table S2.** Relation between average wellbeing or average anxiety-depression and number of surveys completed.

| Number of WB surveys | Number of participants | Mean WB | SE WB | Number of anx/dep surveys | Number of participants | Mean anx/dep | SE anx/dep |
| --- | --- | --- | --- | --- | --- | --- | --- |
| 1 | 21868 | 7.69 | 0.01 | 1 | 23819 | 4.55 | 0.03 |
| 2 | 10207 | 7.71 | 0.01 | 2 | 10786 | 4.69 | 0.04 |
| 3 | 7134 | 7.74 | 0.01 | 3 | 6800 | 4.7 | 0.05 |
| 4 | 5009 | 7.79 | 0.01 | 4 | 3931 | 4.97 | 0.07 |
| 5 | 3134 | 7.78 | 0.01 | 5 | 2072 | 5.09 | 0.09 |
| 6 | 803 | 7.72 | 0.03 | 6 | 1073 | 5.37 | 0.13 |
| 7 | 194 | 7.74 | 0.05 | 7 | 468 | 5.08 | 0.2 |
| 8 | 20 | 7.56 | 0.22 | 8 | 283 | 4.97 | 0.24 |
|  |  |  |  | 9 | 111 | 4.43 | 0.38 |
|  |  |  |  |  |  |  |  |
| 4-8 surveys | 9160 | 7.78 | 0.01 | 4-9 surveys | 7938 | 5.05 | 0.05 |

Note: WB=wellbeing, anxdep=anxiety-depression,

**Figure S1.** Relation between average wellbeing (top) and average anxiety-depression (bottom) and number of surveys completed.

Note: To evaluate potential bias due to selective survey completion, we conducted sensitivity analyses. These showed that individuals with slightly higher wellbeing were more likely to complete more surveys (β = 0.02, *p* = 1.09×10⁻¹⁰), and similarly, those with slightly higher anxious-depressed symptoms also tended to complete more surveys (β = 0.11, *p* = 1.06×10⁻¹⁵).

**Measures**

**Table S3.** Overview of all included measures and their details.

| Phenotype | Scale | Nr of items | Response scale | Response options | Example item | Reference |
| --- | --- | --- | --- | --- | --- | --- |
| Anxious/Depressed | ASEBA Adult Self-Report | 18 | 3-point | 0: not true, 1: sometimes true, 2: very/often true | *I am unhappy, sad, or depressed* | Achenbach & Rescorla, 2003 |
| Withdrawn | ASEBA Adult Self-Report | 9 | 3-point | 0: not true, 1: sometimes true, 2: very/often true | *I am not liked by others* | Achenbach & Rescorla, 2003 |
| Somatic Complaints | ASEBA Adult Self-Report | 12 | 3-point | 0: not true, 1: sometimes true, 2: very/often true | *I feel dizzy or lightheaded* | Achenbach & Rescorla, 2003 |
| Aggressive Behavior | ASEBA Adult Self-Report | 15 | 3-point | 0: not true, 1: sometimes true, 2: very/often true | *I get in many fights* | Achenbach & Rescorla, 2003 |
| Rule-breaking Behavior | ASEBA Adult Self-Report | 14 | 3-point | 0: not true, 1: sometimes true, 2: very/often true | *I damage or destroy my things* | Achenbach & Rescorla, 2003 |
| Intrusive Behavior | ASEBA Adult Self-Report | 6 | 3-point | 0: not true, 1: sometimes true, 2: very/often true | *I am louder than others* | Achenbach & Rescorla, 2003 |
| Attention Problems | ASEBA Adult Self-Report | 15 | 3-point | 0: not true, 1: sometimes true, 2: very/often true | *I have trouble sitting still* | Achenbach & Rescorla, 2003 |
| Thought Problems | ASEBA Adult Self-Report | 10 | 3-point | 0: not true, 1: sometimes true, 2: very/often true | *I cannot get my mind off certain thoughts* | Achenbach & Rescorla, 2003 |
| Wellbeing | Cantril's Self-Anchoring Striving Scale | 1 | 10-point | 1: worst possible life – 10: best possible | *Overall, where on the scale would you place your life? A score of 10 means the best life you can imagine. A score of 0 means the worst life you can imagine.* | Cantril, 1965 |
| Stressful life events | Schokverwerkings Inventarisatie Lijst | - | 4 categories | not at all, in the past year, in the past 5 years, or more than 5 years ago | *death of a partner, divorce/end relationship* | van der Velden et al., 1992 |
| Self-esteem | Rosenberg Self-esteem scale | 10 | 4-point | 1: Strongly Agree – 4: Strongly Disagree | *I feel that I have a number of good qualities* | Rosenberg, 1979 |
| Neuroticism | NEO Five-Factor Inventory | 12 | 5-point | 1: Strongly disagree – 5: Strongly agree | *I often feel tense and jittery* | Costa and McCrae 1992 |
| Loneliness | short scale for loneliness | 3 | 3-point | 1: hardly ever, 2: some of the time, 3: often | *I feel isolated* | Distel et al., 2010; Hughes et al., 2004) |
| Self-rated health | - | 1 | 5-point | 1: bad, 2: mediocre, 3: reasonable, 4: good, 5: excellent | *In general, how would you rate your health?* | - |
| Exercise | - | 1 | binary | yes/no | *Do you exercise regularly?* | - |
| Social support | General Functioning subscale of the Family Assessment Device (FAD) | 12 | 4-point | 1: Strong agreement – 4: Strong disagreement | *In times of crisis we can turn to each other for support.* | Moos & Moos, 1981 |
|  | Duke–UNC Functional Social Support Questionnaire (FSSQ) | 8 | 5-point | 1: much less than I would like – 5: as much as I would like | *I have people who care what happens in my life* | Broadhead et al., 1988 |
| Optimism | revised Life Orientation Test (LOT-R) | 10 | 5-point | 1: Strongly disagree – 5: Strongly agree | *In uncertain times, I usually expect the best* | Scheier et al. 1994 |

**Percentages of Enduring Mental Health**

**Table S4.** Numbers and percentages of participants that show Enduring Mental Health, 1 time mental health problems and/or low wellbeing, or 2 or more times mental health problems and/or low wellbeing, using a **strict** mental health **threshold**.

|  | Anx-Dep | Aggr | Att | Intrusive | Rbb | Somatic | Thought | Withd |  | Any mental health probl | WB | Probl + WB |
| --- | --- | --- | --- | --- | --- | --- | --- | --- | --- | --- | --- | --- |
| EMH | 7098 | 3629 | 3337 | 2366 | 3603 | 3403 | 3522 | 3566 |  | 2695 | 7947 | 1895 |
| 1x problems | 426 | 109 | 285 | 97 | 114 | 177 | 187 | 143 |  | 732 | 482 | 500 |
| 2+ problems | 256 | 11 | 122 | 29 | 27 | 57 | 65 | 59 |  | 373 | 157 | 267 |
|  |  |  |  |  |  |  |  |  |  |  |  |  |
| EMH % | 91% | 97% | 89% | 95% | 96% | 94% | 93% | 95% |  | 71% | 93% | 71% |

Note: EMH=Enduring Mental health, Anx-Dep=Anxiety-Depression, Aggr=Aggression, Att= Attention problems, Intrusive= Intrusive behavior, Rbb= Rule Breaking Behavior, Somatic= Somatic problems, Thought= Thought problems, Withd= Withdrawn behavior, WB=wellbeing.

*This table presents the number and percentage of participants classified into three groups: (1) Enduring Mental Health (EMH), (2) mental health problems and/or low wellbeing at one assessment, and (3) mental health problems and/or low wellbeing at two or more assessments, using the strict threshold. Results are shown separately for each of the eight syndrome scales, for wellbeing, for overall mental health problems, and for the combined measure of mental health problems and wellbeing. For the individual syndrome scales, participants were classified based on whether they exceeded the strict threshold (T-score > 69) on that specific scale across assessments. The overall mental health problems variable reflects whether participants exceeded the strict threshold on* ***any*** *of the eight syndrome scales across assessments. Therefore, prevalence estimates for overall mental health problems are not expected to match those of any single syndrome scale.*

**Table S5.** Cross-tabulation of low wellbeing and mental health problems across assessments, using the strict threshold.

|  | | 0x mental health problems | 1x mental health problems | 2+x mental health problems | | | **Total** |
| --- | --- | --- | --- | --- | --- | --- | --- |
| 0x low wellbeing | | 1895 | 389 | 145 |  | 2429 | |
| 1x low wellbeing | | 0 | 111 | 62 |  | 173 | |
| 2x low wellbeing | | 0 | 0 | 60 |  | 60 | |
| **Total** | 1895 | | 500 | 267 |  | 2662 | |

*This table shows how often participants reported low wellbeing and mental health problems across assessments, using the strict threshold. “0x” indicates that the participant never met criteria across the study period, “1x” indicates that criteria were met at one assessment, and “2+x” indicates that criteria were met at two or more assessments. Participants in the cell with 0x low wellbeing and/or 0x mental health problems were classified as having Enduring Mental Health (EMH). The cells with 0x low wellbeing and 1x or 2x mental health problems represent participants with enduring wellbeing despite mental health problems. In this group, 22% of participants with enduring wellbeing also had mental health problems at least once ((389 + 145) / 2662).*

**Table S6.** Numbers and percentages of participants that show Enduring Mental Health, 1 time mental health problems and/or low wellbeing, or 2 or more times mental health problems and/or low wellbeing, using a **lenient** mental health **threshold**.

|  | Anx-Dep | Aggr | Att | Intrusive | Rbb | Somatic | Thought | Withd | Total problems | WB | Total + WB |
| --- | --- | --- | --- | --- | --- | --- | --- | --- | --- | --- | --- |
| EMH | 6319 | 3300 | 2875 | 2027 | 3409 | 2854 | 3178 | 3022 | 1602 | 7032 | 1124 |
| 1x problems | 770 | 334 | 509 | 305 | 243 | 479 | 398 | 480 | 1131 | 1041 | 788 |
| 2+ problems | 691 | 115 | 306 | 160 | 92 | 304 | 198 | 266 | 1067 | 513 | 750 |
|  |  |  |  |  |  |  |  |  |  |  |  |
| EMH % | 81% | 88% | 78% | 81% | 91% | 78% | 84% | 80% | 42% | 82% | 42% |

Note: EMH=Enduring Mental health, Anx-Dep=Anxiety-Depression, Aggr=Aggression, Att= Attention problems, Intrusive= Intrusive behavior, Rbb= Rule Breaking Behavior, Somatic= Somatic problems, Thought= Thought problems, Withd= Withdrawn behavior, WB=wellbeing.

*This table presents the number and percentage of participants classified into three groups: (1) Enduring Mental Health (EMH), (2) mental health problems and/or low wellbeing at one assessment, and (3) mental health problems and/or low wellbeing at two or more assessments, using the lenient threshold. Results are shown separately for each of the eight syndrome scales, for wellbeing, for overall mental health problems, and for the combined measure of mental health problems and wellbeing. For the individual syndrome scales, participants were classified based on whether they exceeded the lenient threshold (T-score > 65) on that specific scale across assessments. The overall mental health problems variable reflects whether participants exceeded the lenient threshold on* ***any*** *of the eight syndrome scales across assessments. Therefore, prevalence estimates for overall mental health problems are not expected to match those of any single syndrome scale.*

**Table S7.** Cross-tabulation of low wellbeing and mental health problems across assessments, using more lenient mental health threshold.

|  | | 0x mental health problems | 1x mental health problems | 2 mental health problems | | | **Total** |
| --- | --- | --- | --- | --- | --- | --- | --- |
| 0x low wellbeing | | 1124 | 588 | 383 |  | 2095 | |
| 1x low wellbeing | | 0 | 200 | 173 |  | 373 | |
| 2x low wellbeing | | 0 | 0 | 194 |  | 194 | |
| **Total** | 1124 | | 788 | 750 |  | 2662 | |

*This table shows how often participants reported low wellbeing and mental health problems across assessments, using the more lenient threshold. “0x” indicates that the participant never met criteria across the study period, “1x” indicates that criteria were met at one assessment, and “2+x” indicates that criteria were met at two or more assessments. Participants in the cell with 0x low wellbeing and/or 0x mental health problems were classified as having Enduring Mental Health (EMH). The cells with 0x low wellbeing and 1x or 2x mental health problems represent participants with enduring wellbeing despite mental health problems. In this group, 46% of participants with enduring wellbeing also had mental health problems at least once ((588 + 383) / 2662)-.*

**Twin models**

**Table S8.** Twin correlations.

| **Strict** | MZ | DZ | **Lenient** | MZ | DZ |
| --- | --- | --- | --- | --- | --- |
| MHprob+WB | .33 | .14 | MHprob+WB | .29 | .19 |
| Problems | .33 | .10 | Problems | .31 | .12 |
| Wellbeing | .24 | .11 | Wellbeing | .24 | .13 |

**Note:** Mhprobl= mental health problems, WB=wellbeing

**Table S9.** Model comparisons of the univariate binary twin models.

| **Strict** |  |  |  |  |  |  |  |  |  |  |
| --- | --- | --- | --- | --- | --- | --- | --- | --- | --- | --- |
| **MHprob+WB** |  | base | comparison | ep | -2LL | df | AIC | diffLL | diffdf | p |
|  | 1 | ACE |  | 4 | 2137.46 | 1792 | -1446.54 | NA | NA | NA |
|  | 2 | ACE | **AE** | 3 | 2137.47 | 1793 | -1448.53 | 0.01 | 1 | **0.906** |
|  | 3 | ACE | CE | 3 | 2140.82 | 1793 | -1445.18 | 3.36 | 1 | 0.067 |
|  | 4 | ACE | E | 2 | 2173.16 | 1794 | -1414.84 | 35.70 | 2 | 1.77E-08 |
| **Problems** |  | base | comparison | ep | -2LL | df | AIC | diffLL | diffdf | p |
|  | 1 | ACE |  | 4 | 3139.56 | 2632 | -2124.44 | NA | NA | NA |
|  | 2 | ACE | **AE** | 3 | 3140.33 | 2633 | -2125.67 | 0.77 | 1 | **0.381** |
|  | 3 | ACE | CE | 3 | 3150.01 | 2633 | -2115.99 | 10.45 | 1 | 0.001 |
|  | 4 | ACE | E | 2 | 3201.26 | 2634 | -2066.74 | 61.70 | 2 | 3.99E-14 |
| **Wellbeing** |  | base | comparison | ep | -2LL | df | AIC | diffLL | diffdf | p |
|  | 1 | ACE |  | 4 | 2178.86 | 3846 | -5513.14 | NA | NA | NA |
|  | 2 | ACE | **AE** | 3 | 2178.91 | 3847 | -5515.09 | 0.05 | 1 | **0.824** |
|  | 3 | ACE | CE | 3 | 2184.12 | 3847 | -5509.88 | 5.26 | 1 | 0.022 |
|  | 4 | ACE | E | 2 | 2214.05 | 3848 | -5481.95 | 35.19 | 2 | 2.28E-08 |

| **Lenient** | |  | |  | |  | |  | |  | |  | |  | |  | |  | |
| --- | --- | --- | --- | --- | --- | --- | --- | --- | --- | --- | --- | --- | --- | --- | --- | --- | --- | --- | --- |
| **MHprob+WB** |  | base | comparison | | ep | | -2LL | | df | | AIC | | diffLL | | diffdf | | p | |  |
|  | 1 | ACE |  | | 4 | | 2424.47 | | 1792 | | -1159.53 | | NA | | NA | | NA | |  |
|  | 2 | ACE | **AE** | | 3 | | 2424.84 | | 1793 | | -1161.16 | | 0.37 | | 1 | | **0.546** | |  |
|  | 3 | ACE | CE | | 3 | | 2425.69 | | 1793 | | -1160.31 | | 1.22 | | 1 | | 0.269 | |  |
|  | 4 | ACE | E | | 2 | | 2457.01 | | 1794 | | -1130.99 | | 32.54 | | 2 | | 8.57E-08 | |  |
| **Problems** |  | base | comparison | | ep | | -2LL | | df | | AIC | | diffLL | | diffdf | | p | |  |
|  | 1 | ACE |  | | 4 | | 3546.21 | | 2632 | | -1717.79 | | NA | | NA | | NA | |  |
|  | 2 | ACE | **AE** | | 3 | | 3546.72 | | 2633 | | -1719.29 | | 0.51 | | 1 | | **0.477** | |  |
|  | 3 | ACE | CE | | 3 | | 3555.58 | | 2633 | | -1710.42 | | 9.38 | | 1 | | 0.002 | |  |
|  | 4 | ACE | E | | 2 | | 3603.82 | | 2634 | | -1664.18 | | 57.61 | | 2 | | 3.09E-13 | |  |
| **Wellbeing** |  | base | comparison | | ep | | -2LL | | df | | AIC | | diffLL | | diffdf | | p | |  |
|  | 1 | ACE |  | | 4 | | 3302.36 | | 3698 | | -4093.64 | | NA | | NA | | NA | |  |
|  | 2 | ACE | **AE** | | 3 | | 3302.36 | | 3699 | | -4095.64 | | 0.00 | | 1 | | **0.981** | |  |
|  | 3 | ACE | CE | | 3 | | 3307.45 | | 3699 | | -4090.55 | | 5.09 | | 1 | | 0.024 | |  |
|  | 4 | ACE | E | | 2 | | 3348.43 | | 3700 | | -4051.57 | | 46.07 | | 2 | | 9.89E-11 | |  |

Model fit statistics are shown for the ACE model and nested submodels (AE, CE, and E) for the combined measure of mental health problems and wellbeing (MHprob+WB), mental health problems only (Problems), and wellbeing only (Wellbeing). The ACE model includes additive genetic (A), shared environmental (C), and unique environmental (E) influences. The AE, CE, and E models are nested within the ACE model and were compared with the ACE model using likelihood-ratio tests. *ep* = estimated parameters; *−2LL* = minus 2 log likelihood; *df* = degrees of freedom; *AIC* = Akaike Information Criterion; *diffLL* = difference in −2 log likelihood relative to the base model; *diffdf* = difference in degrees of freedom; *p* = p-value for the likelihood-ratio test. MHprob = mental health problems; WB = wellbeing.

**Table S10.** Standardized estimates of additive genetic (A) and unique environmental (E) effects on Enduring Mental Health.

|  | Mental health problems and wellbeing | | Mental health problems | | Wellbeing | |
| --- | --- | --- | --- | --- | --- | --- |
| **Strict** | Estimate | 95%CI | Estimate | 95%CI | Estimate | 95%CI |
| A | 0.51 | .35-.64 | 0.5 | .38-.60 | 0.61 | .43-.75 |
| E | 0.49 | .36-.65 | 0.5 | .40-.62 | 0.39 | .25-.57 |
| **Lenient** | Estimate | 95%CI | Estimate | 95%CI | Estimate | 95%CI |
| A | 0.46 | .31-.59 | 0.46 | .35-.56 | 0.52 | .38-.63 |
| E | 0.54 | .41-.69 | 0.54 | .44-.65 | 0.48 | .37-.62 |

This table presents the standardized variance components from the best-fitting AE twin models for EMH defined using the strict and lenient thresholds. Estimates are shown for the combined EMH measure (mental health problems and wellbeing), mental health problems only, and wellbeing only. A = additive genetic influences; E = unique environmental influences, including measurement error. 95% CI = 95% confidence interval.

**Figure S2.** Standardized estimates of additive genetic (A) and unique environmental (E) effects on Enduring Mental Health.

This figure shows the standardized estimates from Table S9 for additive genetic (A) and unique environmental (E) influences on EMH, shown separately for the combined EMH definition (mental health problems and wellbeing), mental health problems only, and wellbeing only, under the strict (clinical) and lenient (borderline) thresholds.

**Table S11.** Model fit and standardized estimates of additive genetic (A) and unique environmental (E) effects of the bivariate binary twin models with EMH based on problems and EMH based on wellbeing.

| **Strict** | | |  |  | |  | |  | | |  | | |  | |  | |  | |  |
| --- | --- | --- | --- | --- | --- | --- | --- | --- | --- | --- | --- | --- | --- | --- | --- | --- | --- | --- | --- | --- |
| **MHprob vs WB** | | | base | comp | | ep | | -2LL | | | df | | | AIC | | diffLL | | diffdf | | p |
|  | 1 | | ACE |  | 11 | | 4712.62 | | | 6475 | | -8237.38 | | | NA | | NA | | NA | |
|  | 2 | | ACE | **AE** | 8 | | 4581/28 | | | 6478 | | -8374.72 | | | -131.34 | | 3 | | **1** | |
|  | | A | |  | | E | | |  | | | |  |  |  |  |  |  |  |  |
|  | | Problems | | Wellbeing | | Problems | | | Wellbeing | | | |  |  |  |  |  |  |  |  |
| Problems | | .47 (.40-.56) | |  | | .53 (.44-.60) | | |  | | | |  |  |  |  |  |  |  |  |
| Wellbeing | | .42 (.33-.50) | | .42 (.25-.52) | | .58 (.50-.67) | | | .58 (.48-.73) | | | |  |  |  |  |  |  |  |  |
|  | |  | |  | |  | | |  | | | |  |  |  |  |  |  |  |  |
| Correlation | | .92 | |  | | .99 | | |  | | | |  |  |  |  |  |  |  |  |
| **Lenient** | | |  |  | |  | |  | | |  | | |  | |  | |  | |  |
| **MHprob vs WB** | | | base | comp | | ep | | -2LL | | | df | | | AIC | | diffLL | | diffdf | | p |
|  | 1 | | ACE |  | 11 | | 6259 | | | 6327 | | -6394,67 | | | NA | | NA | | NA | |
|  | 2 | | ACE | **AE** | 8 | | 6205 | | | 6330 | | -6454,25 | | | -53,57 | | 3 | | **1** | |
|  | | A | |  | | E | | |  | | | |  |  |  |  |  |  |  |  |
|  | | Problems | | Wellbeing | | Problems | | | Wellbeing | | | |  |  |  |  |  |  |  |  |
| Problems | | .42 | |  | | .58 | | |  | | | |  |  |  |  |  |  |  |  |
| Wellbeing | | .37 | | .40 | | .63 | | | .60 | | | |  |  |  |  |  |  |  |  |
|  | |  | |  | |  | | |  | | | |  |  |  |  |  |  |  |  |
| Correlation | | .80 | |  | | .97 | | |  | | | |  |  |  |  |  |  |  |  |

**Correlates of Enduring Mental Health**

**Table S12.** Odds ratios (ORs) and 95% confidence intervals (CIs) for phenotypic and polygenic correlates of Enduring Mental Health (EMH) using the strict threshold, based on separate univariable logistic regression models.

| Predictor | OR wellbeing | Lower CI wellbeing | Upper CI wellbeing | OR problems | Lower CI problems | Upper CI problems | OR combined | Lower CI combined | Upper CI combined |
| --- | --- | --- | --- | --- | --- | --- | --- | --- | --- |
| **Phenotypic** |  |  |  |  |  |  |  |  |  |
| Sex (female vs male) | **0.75** | 0.63 | 0.90 | **0.66** | 0.56 | 0.78 | **0.75** | 0.62 | 0.92 |
| Year of birth | **0.76** | 0.70 | 0.83 | 1.01 | 0.90 | 1.13 | 1.02 | 0.90 | 1.15 |
| Educational attainment | 1.10 | 1.00 | 1.21 | **1.16** | 1.05 | 1.27 | 1.08 | 0.95 | 1.23 |
| N stressful life events (adolescence) | **0.83** | 0.73 | 0.95 | **0.71** | 0.62 | 0.80 | **0.71** | 0.63 | 0.81 |
| N stressful life events (adulthood) | **0.68** | 0.62 | 0.75 | **0.63** | 0.59 | 0.68 | **0.63** | 0.58 | 0.69 |
| Neuroticism (adult) | **0.31** | 0.28 | 0.34 | **0.35** | 0.32 | 0.38 | **0.34** | 0.31 | 0.38 |
| Optimism (adolescence) | **2.4** | 2.03 | 2.85 | **2.07** | 1.71 | 2.52 | **2.07** | 1.71 | 2.52 |
| Optimism (adult) | **2.86** | 2.55 | 3.22 | **1.93** | 1.74 | 2.14 | **1.90** | 1.71 | 2.11 |
| Self-rated health (adolescence) | **1.58** | 1.40 | 1.78 | **1.49** | 1.31 | 1.70 | **1.49** | 1.31 | 1.70 |
| Self-rated health (adult) | **1.81** | 1.68 | 1.95 | **1.65** | 1.52 | 1.78 | **1.68** | 1.52 | 1.85 |
| Loneliness (adolescence) | **0.39** | 0.33 | 0.45 | **0.38** | 0.31 | 0.46 | **0.38** | 0.32 | 0.46 |
| Loneliness (adult) | **0.45** | 0.43 | 0.48 | **0.49** | 0.46 | 0.53 | **0.48** | 0.44 | 0.52 |
| Exercise (adolescence) | **1.24** | 1.11 | 1.39 | 1.10 | 0.97 | 1.24 | 1.10 | 0.97 | 1.25 |
| Exercise (adult) | **1.19** | 1.10 | 1.29 | **1.21** | 1.13 | 1.30 | **1.25** | 1.14 | 1.36 |
| Social support (adolescence) | **1.65** | 1.45 | 1.87 | **1.51** | 1.31 | 1.73 | **1.51** | 1.31 | 1.73 |
| Social support (adult) | **2.19** | 2.04 | 2.35 | **1.65** | 1.52 | 1.79 | **1.69** | 1.53 | 1.86 |
| **Polygenic scores** |  |  |  |  |  |  |  |  |  |
| ADHD | 0.95 | 0.85 | 1.05 | 0.95 | 0.87 | 1.04 | 0.94 | 0.85 | 1.04 |
| Aggression | 1.06 | 0.96 | 1.17 | 1.03 | 0.95 | 1.13 | 1.05 | 0.95 | 1.16 |
| Antisocial Behavior | 1.00 | 0.90 | 1.10 | 0.95 | 0.87 | 1.03 | 0.92 | 0.83 | 1.01 |
| Anxiety | **0.85** | 0.77 | 0.95 | **0.81** | 0.75 | 0.88 | **0.82** | 0.75 | 0.90 |
| Autism Spectrum Disorder | 0.96 | 0.87 | 1.06 | 0.91 | 0.84 | 1.00 | 0.91 | 0.82 | 1.00 |
| Bipolar disorder | 0.95 | 0.85 | 1.06 | **0.90** | 0.83 | 0.99 | 0.91 | 0.82 | 1.01 |
| Childhood Maltreatment | 0.86 | 0.78 | 0.95 | **0.86** | 0.79 | 0.93 | **0.80** | 0.72 | 0.88 |
| Educational Attainment | 1.03 | 0.93 | 1.14 | 1.05 | 0.97 | 1.15 | 0.98 | 0.88 | 1.08 |
| Loneliness | 0.84 | 0.76 | 0.93 | **0.86** | 0.80 | 0.94 | **0.84** | 0.76 | 0.93 |
| Major Depressive Disorder | 0.81 | 0.73 | 0.9 | **0.76** | 0.7 | 0.83 | **0.80** | 0.72 | 0.88 |
| Neuroticism | 0.85 | 0.77 | 0.94 | **0.81** | 0.75 | 0.89 | **0.84** | 0.76 | 0.92 |
| Post-traumatic stress disorder | 0.94 | 0.84 | 1.05 | **0.80** | 0.73 | 0.87 | **0.78** | 0.7 | 0.87 |
| Risky Behavior | 1.08 | 0.97 | 1.20 | 0.98 | 0.90 | 1.07 | 0.97 | 0.87 | 1.07 |
| Schizophrenia | 0.89 | 0.79 | 0.99 | **0.89** | 0.81 | 0.98 | **0.88** | 0.79 | 0.98 |
| Wellbeing | 1.31 | 1.19 | 1.46 | **1.27** | 1.16 | 1.38 | **1.23** | 1.11 | 1.37 |

This table presents ORs and 95% CIs from separate univariable logistic regression models examining the associations between each predictor and EMH under the strict threshold. Results are shown separately for enduring wellbeing, enduring absence of mental health problems, and the combined EMH definition. ORs greater than 1 indicate that higher values of the predictor are associated with higher odds of EMH, whereas ORs less than 1 indicate lower odds of EMH. For sex, the OR compares females with males.

**Table S13.** Odds ratios (ORs) and 95% confidence intervals (CIs) for phenotypic and polygenic correlates of Enduring Mental Health (EMH) using the lenient threshold, based on separate univariable logistic regression models.

| Predictor | **OR wellbeing** | Lower CI wellbeing | Upper CI wellbeing | **OR problems** | Lower CI problems | Upper CI problems | **OR combined** | Lower CI combined | Upper CI combined |
| --- | --- | --- | --- | --- | --- | --- | --- | --- | --- |
| **Phenotypic** |  |  |  |  |  |  |  |  |  |
| Sex (female vs male) | **0.75** | 0.66 | 0.85 | **0.61** | 0.53 | 0.71 | **0.72** | 0.60 | 0.86 |
| Year of birth | **0.77** | 0.72 | 0.81 | 1.07 | 0.96 | 1.18 | 1.08 | 0.96 | 1.21 |
| Educational attainment | **1.12** | 1.05 | 1.20 | **1.17** | 1.07 | 1.28 | **1.19** | 1.05 | 1.34 |
| N stressful life events (adolescence) | **0.77** | 0.71 | 0.84 | **0.73** | 0.64 | 0.82 | **0.73** | 0.65 | 0.82 |
| N stressful life events (adulthood) | **0.73** | 0.69 | 0.78 | **0.68** | 0.64 | 0.73 | **0.66** | 0.61 | 0.72 |
| Neuroticism (adult) | **0.34** | 0.32 | 0.36 | **0.34** | 0.32 | 0.37 | **0.35** | 0.32 | 0.39 |
| Optimism (adolescence) | **2.18** | 1.92 | 2.47 | **2.08** | 1.71 | 2.53 | **2.08** | 1.71 | 2.53 |
| Optimism (adult) | **2.74** | 2.50 | 3.00 | **2.03** | 1.83 | 2.24 | **2.04** | 1.84 | 2.26 |
| Self-rated health (adolescence) | **1.57** | 1.44 | 1.72 | **1.66** | 1.47 | 1.88 | **1.66** | 1.46 | 1.87 |
| Self-rated health (adult) | **1.69** | 1.59 | 1.79 | **1.64** | 1.52 | 1.77 | **1.74** | 1.59 | 1.90 |
| Loneliness (adolescence) | **0.47** | 0.42 | 0.53 | **0.41** | 0.33 | 0.51 | **0.41** | 0.34 | 0.51 |
| Loneliness (adult) | **0.45** | 0.43 | 0.47 | **0.49** | 0.45 | 0.53 | **0.47** | 0.43 | 0.52 |
| Exercise (adolescence) | **1.13** | 1.04 | 1.22 | **1.22** | 1.08 | 1.37 | **1.22** | 1.08 | 1.37 |
| Exercise (adult) | **1.17** | 1.11 | 1.24 | **1.20** | 1.12 | 1.28 | **1.23** | 1.13 | 1.34 |
| Social support (adolescence) | **1.65** | 1.49 | 1.82 | **1.50** | 1.32 | 1.71 | **1.50** | 1.32 | 1.70 |
| Social support (adult) | **2.26** | 2.12 | 2.40 | **1.84** | 1.66 | 2.03 | **1.98** | 1.76 | 2.24 |
| **Polygenic scores** |  |  |  |  |  |  |  |  |  |
| ADHD | **0.91** | 0.84 | 0.98 | **0.90** | 0.84 | 0.98 | **0.90** | 0.82 | 0.99 |
| Aggression | 1.00 | 0.94 | 1.07 | 0.97 | 0.90 | 1.05 | 0.95 | 0.86 | 1.04 |
| Antisocial Behavior | 0.99 | 0.92 | 1.07 | 1.00 | 0.92 | 1.08 | 0.97 | 0.88 | 1.06 |
| Anxiety | **0.88** | 0.82 | 0.94 | **0.87** | 0.80 | 0.94 | **0.89** | 0.82 | 0.98 |
| Autism Spectrum Disorder | **0.92** | 0.86 | 0.99 | 0.94 | 0.87 | 1.01 | 0.94 | 0.86 | 1.03 |
| Bipolar disorder | **0.89** | 0.83 | 0.96 | 0.92 | 0.85 | 1.00 | 0.93 | 0.84 | 1.02 |
| Childhood Maltreatment | **0.87** | 0.81 | 0.93 | **0.89** | 0.83 | 0.96 | **0.84** | 0.77 | 0.93 |
| Educational Attainment | 1.04 | 0.97 | 1.12 | 1.03 | 0.96 | 1.12 | 1.03 | 0.94 | 1.13 |
| Loneliness | **0.85** | 0.79 | 0.91 | **0.90** | 0.83 | 0.97 | **0.85** | 0.77 | 0.93 |
| Major Depressive Disorder | **0.83** | 0.77 | 0.89 | **0.78** | 0.72 | 0.85 | **0.81** | 0.74 | 0.89 |
| Neuroticism | **0.88** | 0.82 | 0.94 | **0.84** | 0.78 | 0.90 | **0.81** | 0.74 | 0.89 |
| Post-traumatic stress disorder | **0.90** | 0.84 | 0.97 | **0.82** | 0.75 | 0.89 | **0.80** | 0.72 | 0.88 |
| Risky Behavior | 1.02 | 0.95 | 1.10 | 1.01 | 0.94 | 1.09 | 1.01 | 0.92 | 1.10 |
| Schizophrenia | **0.92** | 0.85 | 0.99 | 0.95 | 0.87 | 1.03 | 0.93 | 0.84 | 1.02 |
| Wellbeing | 1.23 | 1.15 | 1.32 | **1.23** | 1.14 | 1.33 | 1.23 | 1.12 | 1.35 |

This table presents ORs and 95% CIs from separate univariable logistic regression models examining the associations between each predictor and EMH under the lenient threshold. Results are shown separately for enduring wellbeing, enduring absence of mental health problems, and the combined EMH definition. ORs greater than 1 indicate that higher values of the predictor are associated with higher odds of EMH, whereas ORs less than 1 indicate lower odds of EMH. For sex, the OR compares females with males.

**Figure S3.** Comparison of phenotypic correlates of Enduring Mental Health based on strict versus lenient mental health thresholds.

**Figure S4.** Comparison of polygenic score correlates of Enduring Mental Health based on strict versus lenient mental health thresholds.

**Table S14.** Odds ratios (ORs) and 95% confidence intervals (CIs) for phenotypic and polygenic correlates of Enduring Mental Health (EMH) using the strict threshold, based on the multivariable logistic regression models.

|  | **Adolescent + adult predictor model (n=277)** | | | | **Adult predictor model (n=1275)** | | | |
| --- | --- | --- | --- | --- | --- | --- | --- | --- |
| Predictor | OR | CI lower | CI upper | p-value | OR | CI lower | CI upper | p-value |
| (Intercept) | 1.29 | 0.26 | 6.45 | 0.755 | 2.46 | 1.38 | 4.38 | 0.002 |
| Sex (female vs male) | 1.80 | 0.74 | 4.36 | 0.192 | 1.12 | 0.82 | 1.55 | 0.474 |
| N stressful life events (adolescence) | 0.73 | 0.55 | 0.96 | 0.027 |  |  |  |  |
| N stressful life events (adulthood) | 0.84 | 0.59 | 1.20 | 0.336 | **0.67** | 0.59 | 0.76 | **8.76x10^-10^** |
| Neuroticism (adult) | **0.36** | 0.23 | 0.58 | **2.86x10^-5^** | **0.48** | 0.40 | 0.56 | **9.64 x10^-18^** |
| Optimism (adolescence) | 1.54 | 0.99 | 2.41 | 0.056 |  |  |  |  |
| Optimism (adult) | 0.70 | 0.50 | 0.99 | 0.042 | 1.14 | 0.99 | 1.32 | 0.071 |
| Self-rated health (adolescence) | 1.08 | 0.72 | 1.61 | 0.709 |  |  |  |  |
| Self-rated health (adult) | 1.36 | 0.92 | 2.00 | 0.122 | **1.24** | 1.06 | 1.44 | **0.007** |
| Loneliness (adolescence) | **0.61** | 0.43 | 0.87 | **0.007** |  |  |  |  |
| Loneliness (adult) | 0.96 | 0.64 | 1.44 | 0.844 | **0.79** | 0.68 | 0.92 | **0.003** |
| Exercise (adult) | 0.82 | 0.59 | 1.13 | 0.226 | 1.04 | 0.91 | 1.18 | 0.586 |
| Social support (adolescence) | 1.09 | 0.75 | 1.58 | 0.647 |  |  |  |  |
| Social support (adult) | 1.56 | 0.99 | 2.47 | 0.054 | 1.13 | 0.99 | 1.30 | 0.075 |
| **Polygenic scores** |  |  |  |  |  |  |  |  |
| Childhood Maltreatment | 0.91 | 0.62 | 1.33 | 0.621 | 0.85 | 0.74 | 0.99 | 0.035 |
| Loneliness | 0.97 | 0.66 | 1.42 | 0.879 | 1.07 | 0.91 | 1.25 | 0.436 |
| Major Depressive Disorder | 1.11 | 0.70 | 1.75 | 0.661 | 0.92 | 0.77 | 1.10 | 0.381 |
| Neuroticism | 1.23 | 0.75 | 2.03 | 0.415 | 0.95 | 0.76 | 1.18 | 0.620 |
| Schizophrenia | 0.87 | 0.59 | 1.28 | 0.478 | 0.88 | 0.76 | 1.03 | 0.107 |
| Wellbeing | 1.26 | 0.76 | 2.10 | 0.367 | 0.89 | 0.71 | 1.11 | 0.289 |
| Anxiety | 0.67 | 0.45 | 1.02 | 0.059 | 0.86 | 0.75 | 0.98 | 0.025 |

Odds ratios (ORs), 95% confidence intervals (CIs), and p-values are shown for multivariable logistic regression models predicting the combined EMH definition under the strict threshold. Two models were estimated: an adolescent + adult predictor model (n = 277) and an adult predictor model (n = 1275). All predictors listed within a model were entered simultaneously; thus, each OR reflects the association between that predictor and EMH adjusted for the other predictors in the same model. ORs greater than 1 indicate higher odds of EMH, whereas ORs less than 1 indicate lower odds of EMH. For sex, the OR compares females with males. Blank cells indicate that a predictor was not included in that model.

**Figure S5.** Correlates of the combined Enduring Mental Health (EMH) definition under the strict threshold from the multivariable regression model.

This figure displays odds ratios and 95% confidence intervals from the multivariable logistic regression models predicting the combined EMH definition under the strict threshold, shown separately for the adolescent + adult predictor model and the adult predictor model. (adolescence) and (adult) indicate that the correlate is measured respectively during adolescence and adulthood.
